# Editorial: Advancements in microbial engineering for natural product synthesis

**DOI:** 10.3389/fbioe.2026.1915541

**Published:** 2026-07-23

**Authors:** Cheng Li, Kai Li, Yuanyuan Huang

**Affiliations:** 1 Polynovo Biotech Co., Ltd, Suzhou, China; 2 Department of Biology, Massachusetts Institute of Technology, Cambridge, MA, United States; 3 School of Life Sciences and Biotechnology, Shanghai Jiao Tong University, Shanghai, China; 4 Department of Biomedical Engineering, Columbia University, New York, NY, United States

**Keywords:** genetic engineering, industrial production, microbial cell factories, natural product, renewable carbon sources, sustainability engineering

## Introduction

The transition from petrochemical-dependent chemical synthesis to sustainable biomanufacturing represents a paradigm shift in modern industry. While traditional chemical processes have powered industrial production for decades, their reliance on non-renewable feedstocks, harsh reaction conditions, and toxic byproducts pose growing environmental and economic burdens. In contrast, microbial cell factories offer a transformative solution: by harnessing nature’s enzymatic precision, these living systems can convert renewable carbon sources into high-value natural products under mild conditions. At the core of this revolution lies the principle of atom economy—the efficiency by which every carbon atom in feedstocks is maximized in the final product. Unlike chemical synthesis, which often generates wasteful byproducts, biological pathways are evolutionarily optimized to minimize carbon loss, achieving unparalleled selectivity and resource efficiency. This Research Topic, “Advancements in microbial engineering for natural product synthesis,” showcases how cutting-edge metabolic and genetic engineering are accelerating this transition, with a focus on technologies that enhance industrial scalability and carbon utilization.

## The carbon conundrum: why biology beats chemistry

Industrial biotechnology’s superiority in carbon economy stems from two fundamental advantages: specificity and sustainability. Chemical syntheses frequently require multistep reactions, each generating side products that demand costly purification. In contrast, microbial enzymes catalyze reactions with exquisite substrate specificity, directly funneling carbon atoms into target molecules. For example, enzymatic cascades can achieve >90% carbon yield in assembling complex natural products—a feat unattainable in many chemical processes. Furthermore, biological systems can thrive on diverse carbon substrates, enabling the utilization of non-food feedstocks such as lignocellulose, waste glycerol, or CO_2_. This flexibility not only reduces reliance on fossil resources but also avoids competition with the food supply chain—a critical ethical and economic imperative.

## Engineering for industrial viability: from proof-of-concept to factory floor

This Research Topic’s contributions highlight transformative strategies to bridge laboratory success to industrial-scale production. First, Ou et al. tackled the challenge of precursor scarcity in the industrial workhorse *Corynebacterium glutamicum* by rewiring central carbon metabolism (Ou et al.). By deleting the *iolR* gene and optimizing the phosphoenolpyruvate transport system (PTS), they achieved a remarkable 3-fold increase in shikimate production—a key precursor for the antiviral drug Tamiflu. This study demonstrates how metabolic engineering can unlock high-yield pathways in non-pathogenic, GRAS-qualified hosts, paving the way for safe and scalable biopharmaceutical manufacturing. Second, Wang et al.’s comprehensive review on organic acid production using non-food carbon sources (e.g., agricultural waste) underscored the economic feasibility of lignocellulosic biorefineries (Wang et al.). By engineering microbial transporters and regulatory circuits, they showed how microbes could efficiently convert complex biomass into platform chemicals, directly addressing the cost barrier of sustainable feedstocks. Together, these studies establish a blueprint for carbon-efficient biomanufacturing.

## Expanding the biomanufacturing frontier: beyond chemicals to materials and medicine

The Topic further illustrates how microbial engineering is reshaping diverse industries. Garlapati et al. pioneered a green approach to nanomedicine by repurposing microalgae as photosynthetic factories for metal nanoparticle synthesis (Garlapati et al.). By harnessing sunlight and CO_2_ as energy and carbon inputs, their system offers a sustainable alternative to toxic chemical reduction methods, with applications in cancer diagnostics. Concurrently, Chen and Tang’s analysis of endophytic fungi from Polygonaceae plants revealed a treasure trove of biosynthetic diversity. These microorganisms, which co-evolve with host plants to produce bioactive metabolites, offer a novel pipeline for discovering drug leads and crop stress-resilience enhancers (Chen and Tang). Collectively, these studies showcase how engineered microbes can synthesize not only chemicals but also advanced materials and therapeutics, all while sequestering carbon from waste streams.

## The path forward: scaling sustainability through systems engineering

As we progress, two grand challenges remain: feedstock diversification and process intensification. The current reliance on first-generation sugars (e.g., glucose from corn) is unsustainable. Thus, engineering microbes to ferment lignocellulose or utilize CO_2_ directly—via synthetic pathways like C1 fixation—is paramount. Wang et al.’s work on non-food carbon sources provides critical design principles for such platforms. Additionally, systems biology approaches (e.g., dynamic flux balance analysis) are enabling strain optimization at unprecedented scales. By integrating high-throughput screening, AI-driven pathway prediction, and bioprocess modeling, we can now domesticate microbes to thrive on cheap, abundant, and non-edible carbon substrates while maintaining product titers and yields competitive with chemical industries.

## Conclusion: a green chemical future built on biology

This Research Topic makes a compelling case: the future of industrial synthesis lies not in petrochemical vats but in microbial fermenters. By marrying the atom economy of biology with the precision of synthetic biology, we are creating production systems that are simultaneously economical, sustainable, and carbon-efficient. As non-food feedstocks become the new norm, and as engineered microbes master complex chemistries, biomanufacturing will not only supplant polluting chemical processes—it will redefine what is possible in materials science, medicine, and agriculture. We invite the scientific community to build upon these advancements, accelerating the development of a circular bioeconomy where every carbon atom is valued, recycled, and transformed into solutions for humanity’s greatest challenges.

